# eIF3f reduces tumor growth by directly interrupting clusterin with anti-apoptotic property in cancer cells

**DOI:** 10.18632/oncotarget.8105

**Published:** 2016-03-15

**Authors:** Ji-Yeon Lee, Hyun-Ji Kim, Seung Bae Rho, Seung-Hoon Lee

**Affiliations:** ^1^ Department of Life Science, YongIn University, Samga-dong Chuingu, Yonginsi, Korea; ^2^ Research Institute, National Cancer Center, Ilsandong-gu, Goyang-si Gyeonggi-do, Korea

**Keywords:** clusterin, eukaryotic translation initiation factor 3, subunit f, cancer, apoptosis

## Abstract

Clusterin is a secretory heterodimeric glycoprotein and the overexpression of secretory clusterin (sCLU) promotes cancer cell proliferation and reduces chemosensitivity. Therefore, sCLU might be an effective target for anticancer therapy. In the current study, we identified eIF3f as a novel CLU-interacting protein and demonstrated its novel function as a CLU inhibitor. The overexpression of eIF3f retarded cancer cell growth significantly and induced apoptosis. In addition, eIF3f interacted with the α-chain (1–227) of sCLU. This interaction blocked modification of psCLU, thereby decreasing the expression and secretion of α/β CLU. Consequently, the overexpression of eIF3f suppressed Akt and ERK signaling and subsequently depleted CLU expression. In addition, eIF3F stabilized p53, which increased the expression of p21 and Bax. Interestingly, the expression of Bax was increased without the activation of p53. eIF3f injected into a xenograft model of human cervical cancer in nude mice markedly inhibited tumor growth. The identification of this novel function of eIF3f as a sCLU inhibitor might open novel avenues for developing improved strategies for CLU-targeted anti-cancer therapies.

## INTRODUCTION

Clusterin (CLU), also known as apolipoprotein J (APO J), sulfated glycoprotein (SGP-2), or testosterone repressed prostate message-2 (TRPM-2), is a glycoprotein that acts in a variety of physiological processes such as the cell cycle, DNA repair, tissue remodeling, membrane recycling, lipid transport, immune system regulation, cell adhesion, and apoptosis [1–5]. There are two isoforms of CLU in human cells: secretory CLU (sCLU) and nuclear CLU (nCLU). sCLU is translated into a 60-kDa sCLU precursor protein (psCLU) from the first AUG codon. This protein is then translocated directly into the endoplasmic reticulum (ER) by a leader signal peptide, where it is glycosylated and cleaved into α- and β-chains in the trans-Golgi compartments. Finally, the α- and β-chains are held together by five disulfide bonds in the cytoplasm, and the protein is then secreted from the cell [1, 3]. The nCLU isoform is 55 kDa in size; it is generated initially as a 49-kDa nCLU precursor protein (pnCLU) from the second AUG codon by an alternative splicing event that eliminates exon II, which encodes the first AUG codon and signal peptide. Therefore, α/β cleavage and glycosylation do not occur. pnCLU is located in the cytoplasm normally; it then undergoes glycosylation to form the 55-kDa nCLU, which is translocated from the cytoplasm to the nucleus in response to cell damage [6, 7].

CLU expression is a key factor in tumorigenesis and metastasis. Elevated CLU mRNA and protein levels have been reported in various human cancers, including prostate [8], breast [9], bladder [10], lung [11], renal [12], colon [13], ovarian [14], and cervical [15, 16] cancers. In particular, compared with normal tissues, sCLU and nCLU are overexpressed and downregulated in malignancies. sCLU functions as a pro-survival factor, whereas nCLU has a pro-apoptotic role [17, 18]. For example, in LNCaP prostate cancer cells, sCLU was associated with tumor progression, and it protected cells from the effects of chemotherapeutic drugs [19]. In addition, the knockdown of sCLU in osteosarcoma cells sensitized cells to chemotherapy and oxidative stress signals [20]. Therefore, sCLU is an attractive target for cancer treatment.

In the current study, we clarified the molecular regulation and role of different CLU isoforms in cancer development by performing Y2H screening, which revealed that eIF3f is a CLU-interacting protein. eIF3f is a subunit of the eIF3 complex, which consists of 13 non-identical subunits (eIF3 a-m). Together with eIF1, eIF1A, and eIF2-GTP-Met-tRNAi, eIF3 binds to the 40S ribosomal subunit, which promotes the binding of methionyl-tRNA and mRNA [21–24]. *eIF3f* expression is downregulated in most cancer cells because of the loss of the *eIF3f* allele [25–27]. eIF3f interacts with heterogeneous nuclear ribonucleoprotein (hnRNP) and induces rRNA degradation by interfering with the interaction between hnRNP and rRNA, which subsequently inhibits protein translation [25, 28]. Increased expression of eIF3f reduces cellular growth by inducing apoptosis in melanoma and pancreatic cancer cells [23–25]. In contrast, knocking down eIF3f using siRNA in normal pancreatic HPDE cells increased cell proliferation, migration, and chemotherapeutic resistance [28]. This suggests that eIF3f might be an important negative regulator of cell survival and carcinogenesis. However, the molecular mechanism by which the increased expression of eIF3f induces apoptosis is poorly understood. In the current study, we found that eIF3f induced apoptosis and inhibited tumor growth *in vitro* and *in vivo.* Furthermore, we assessed how eIF3f affects cancer cell growth as well as its relationship with CLU, and revealed the potential of eIF3f as an effective cancer therapeutic target.

## RESULTS

### eIF3f interacts with CLU in the cytoplasm

**Figure 1:**
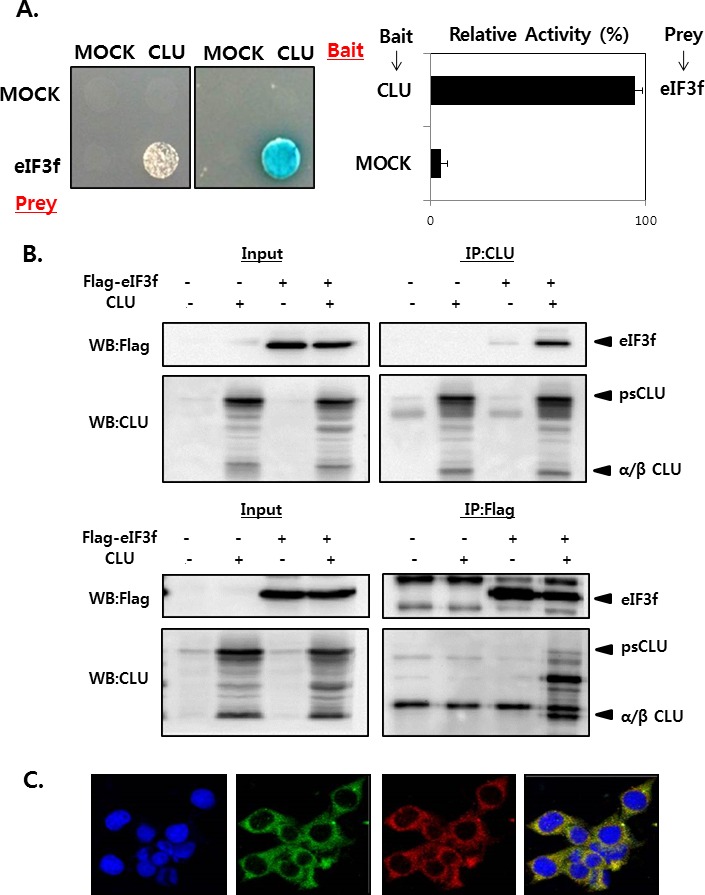
CLU interacts with eIF3f in the cytoplasm **A.** Transformants were tested for their ability to grow on medium lacking leucine (left) and expressing β-galactosidase (right). The interaction between eIF3f and CLU was assessed by relative β-galactosidase expression. **B.** HEK293a cells were transfected with expression vectors encoding the indicated proteins. After 2 days, cell lysates were immunoprecipitated using anti-CLU or -FLAG antibodies and immunoblotted using anti-CLU or -FLAG antibodies. **C.** ICC was performed in HeLa cells using anti-eIF3f and -CLU antibodies, and the nuclei were stained with 4′,6-diamidino-2-phenylindole. Cells were imaged using a confocal fluorescence microscope. The sub-cellular localization of eIF3f (green) and CLU (red) and their co-localization (yellow) are shown (magnification, ×400).

We first performed yeast two-hybrid screening to identify novel CLU-interacting partners in cells. CLU was used as the bait, and a human cDNA library was used as the prey, and the results revealed that eIF3f was a CLU binding partner (data not shown). The interaction between CLU and eIF3f was confirmed by growth assays and Δ-galactosidase assays using a yeast two-hybrid system. Co-transformants of CLU and eIF3f grew only on leucine-deficient plates or appeared blue in color on plates containing x-gal (Figure 1A). To further confirm this observation, their interaction was assessed *in vitro* using co-immunoprecipitation. As shown in Figure 1B, eIF3f was detected in immunoprecipitates using CLU antibodies and vice versa in HEK293a cells. These results suggest that CLU can bind directly to eIF3f. We next assessed the subcellular localization of eIF3f and CLU using immunocytochemistry. Figure 1C shows that eIF3f and CLU were co-localized mainly in the cytoplasm. Therefore, these results suggest that eIF3f and CLU strongly interact with each other in the cytoplasm.

### Expression of eIF3f and CLU

**Figure 2:**
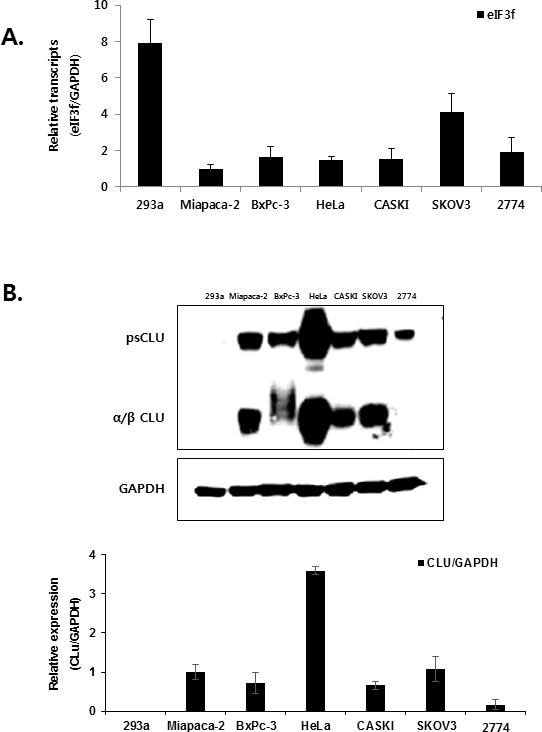
Expression of eIF3f and CLU **A.** Total RNA was isolated from a normal cell line (HEK293a), pancreatic cancer cell lines (Miapaca-2, BxPc-3), cervical cancer cell lines (HeLa, CASKI), and ovarian cancer cell lines (SKOV3, 2774). The expression of *eIF3f* mRNA was then analyzed using qRT-PCR and normalized to glyceraldehyde-3-phosphate dehydrogenase (*GAPDH*) mRNA. **B.** Whole-cell lysates were isolated from the same cancer cell lines described in **A.** and analyzed using Western blotting. The density of the bands was quantified using Image J software, and CLU protein levels were reported as the CLU:GAPDH ratio.

The cell lines used in the present study were selected by examining the expression of eIF3f and CLU. Previous studies revealed that *eIF3f* expression was downregulated in most human tumors compared with normal tissues using a cancer profiling array and qRT-PCR [25]. Therefore, we compared *eIF3f* mRNA levels in six human cancer cell lines (Miapaca-2, BxPc-3, HeLa, CASKI, SKOV3, and 2774) and a normal cell line (HEK293a) using qRT-PCR. Miapaca-2 cells were used as a negative control [25], and *eIF3f* mRNA levels were normalized to *GAPDH.* Consistent with a previous study [25], *eIF3f* mRNA was decreased significantly by 60-80% in cancer cells compared with normal cell line (Figure 2A). In addition, the same cancer cell lines expressed different levels of endogenous CLU protein; among these, HeLa cells had the highest CLU expression (Figure 2B). Therefore, HeLa and BxPc-3 cells were used in subsequent experiments because of their relatively decreased eIF3f expression and increased CLU expression.

### The overexpression of eIF3f inhibits cancer cell growth and induces apoptosis

**Figure 3:**
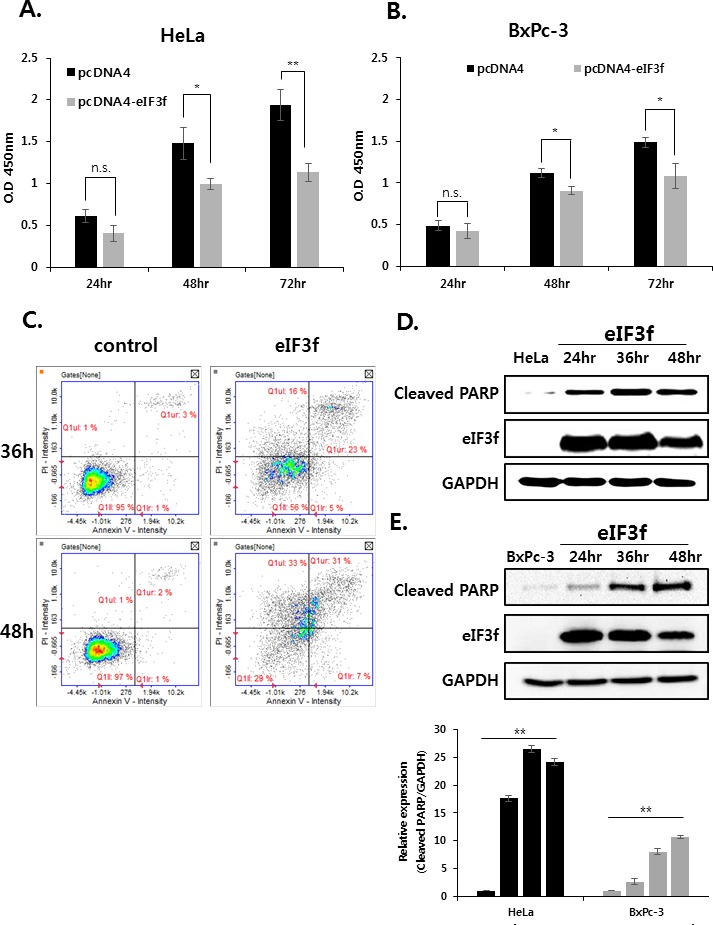

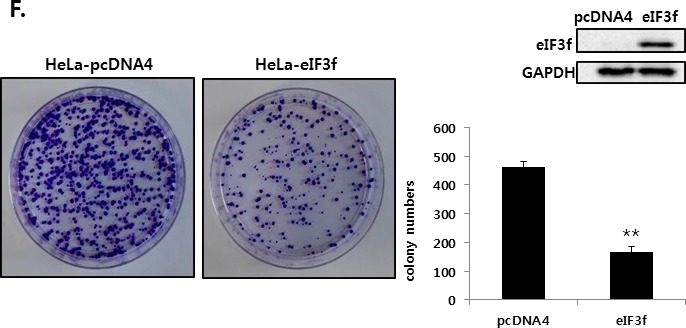
Overexpressed eIF3f inhibits cancer cell proliferation and induces apoptosis The same number of HeLa **A.** and BxPc-3 **B.** cells were plated in triplicate in 96-well plates. The O.D value of cells was then measured at the indicated time points. **C.** HeLa cells were transfected with pcDNA4 or pcDNA4-eIF3f. At the indicated time points, cells were analyzed using flow cytometry. Representative dot plots of Annexin V/PI staining in HeLa cells are shown. HeLa **D.** and BxPc-3 **E.** cells were transfected with pcDNA4-eIF3f and harvested at 24-h intervals. eIF3f or cleaved PARP was then detected using Western blotting. **F.** One thousand HeLa cells transfected with control pcDNA4 or pcDNA4-eIF3f were re-seeded in 100-mm cell culture dishes and incubated for 2 weeks to allow colony formation. The media were then removed, and colonies were stained with 5% crystal violet. The plates were rinsed with water, and the total colony numbers were counted. The same cell lysates were also used to evaluate eIF3f expression using Western blotting.

Previously, qRT-PCR experiments revealed that eIF3f was downregulated significantly in cancer cell lines (Figure 2A). This suggests that decreased eIF3f expression might play a crucial role in tumorigenesis. Therefore, we next evaluated the effect of eIF3f on cancer cell growth by transfecting HeLa and BxPc-3 cells with empty vector or an eIF3f expression vector, and then monitored the growth rates for 24-72 h. Data revealed that eIF3f transfection retarded cell growth compared with control cells in a time-dependent manner. The eIF3f-induced growth inhibition was ~40% more effective in HeLa than BxPc-3 cells after 72 h (Figure 3A, 3B). However, other cell lines that express only low levels of CLU exhibited relatively low growth inhibition in the presence of eIF3f (data not shown). We then examined whether the inhibited cell growth induced by eIF3f overexpression was associated with increased apoptosis using flow cytometry. Data revealed that apoptosis was increased significantly in HeLa cells transfected with eIF3f compared with empty vector alone (Figure 3C). To confirm these observations, cleaved PARP, which is a marker of apoptosis, was analyzed by Western blotting. Data revealed that the overexpression of eIF3f increased cleaved PARP levels in a time-dependent manner (Figure 3D, 3E). These results suggest that the overexpression of eIF3f induces apoptosis in cancer cells, and that the reduced number of eIF3f-transfected cells was due to an increased level of spontaneous apoptosis.

We further compared the ability of these cell lines to form colonies. Consistent with the cell growth data, eIF3f-transfected HeLa cells formed significantly fewer colonies than did vector-transfected cells. The expression of eIF3f was confirmed by Western blotting (Figure 3F). Therefore, eIF3f reduced cancer cell proliferation significantly.

### eIF3f interacts with the α-chain of sCLU

**Figure 4:**
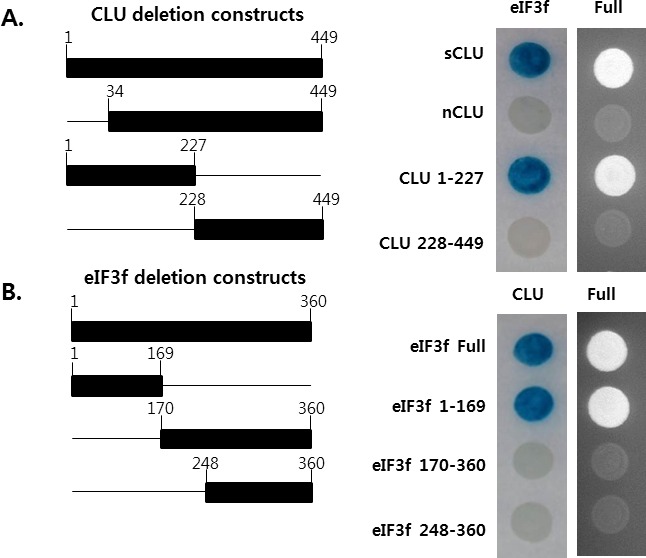
Mapping of the regions mediating the interaction between eIF3f and CLU The left schematic diagram indicates the cDNA deletion constructs of CLU and eIF3f. The right panel shows the results of growth and β-galactosidase assays using yeast two-hybrid assays.

To identify the specific region responsible for the interaction between CLU and eIF3f, we generated four deletion constructs for CLU and eIF3f each and performed yeast two-hybrid assays. As shown in Figure 4, the region between amino acids 1 and 227 of CLU interacts directly with eIF3f, whereas the region between amino acids 1 and 169 of eIF3f interacts directly with CLU and was phosphorylated by CDK11p46 during apoptosis [29, 30] (Figure 4A, 4B). These results suggest that eIF3f might affect the function of CLU by interacting with the α-chain of sCLU (1-227 amino acids).

### eIF3f reduces sCLU expression and secretion

**Figure 5:**
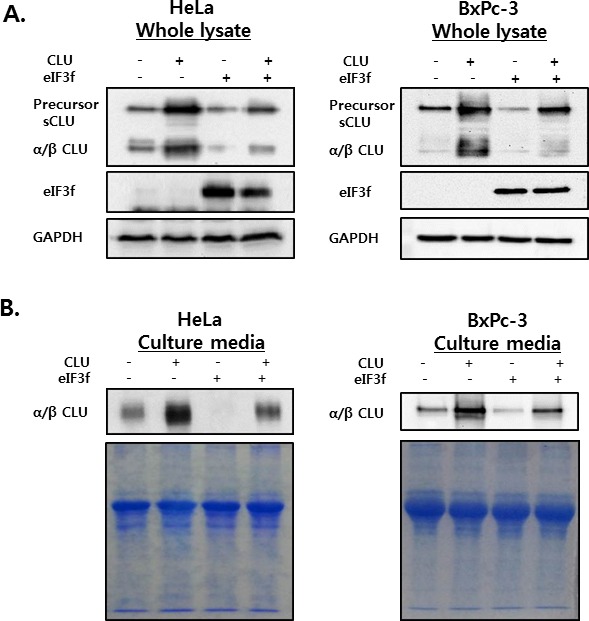
sCLU expression and secretion in whole cell lysates and culture media HeLa and BxPc-3 cells were transfected with CLU or eIF3f expression vectors. Forty-eight hours after transfection, whole cell lysates **A.** and culture media **B.** were harvested. The expression and secretion of sCLU were detected using Western blotting. GAPDH blots and Coomassie staining confirmed equal protein loading.

To investigate the effect of eIF3f on intracellular and extracellular sCLU, whole cell lysates and culture media were isolated from HeLa and BxPc-3 cells transfected with CLU or eIF3f and then analyzed by Western blotting using CLU antibodies. High levels of psCLU and α/β CLU were detected in whole cell lysates of CLU-transfected cells. In contrast, eIF3f-transfected cells expressed decreased levels of both psCLU and α/β CLU. In addition, co-transfection with CLU and eIF3f resulted in a depletion of sCLU expression compared with transfection with CLU only (Figure 5A).

The secretion of sCLU was confirmed in culture medium from cells treated under the same conditions. Similarly, the overexpression of CLU increased the secretion of sCLU, whereas the overexpression of eIF3f decreased the secretion of sCLU significantly (Figure 5B). These results suggest that eIF3f might interact with CLU protein to reduce the expression of sCLU in both intracellular and extracellular compartments by preventing structural changes.

### eIF3f overexpression activates p53 and Bax and inhibits Akt and ERK signaling

**Figure 6:**
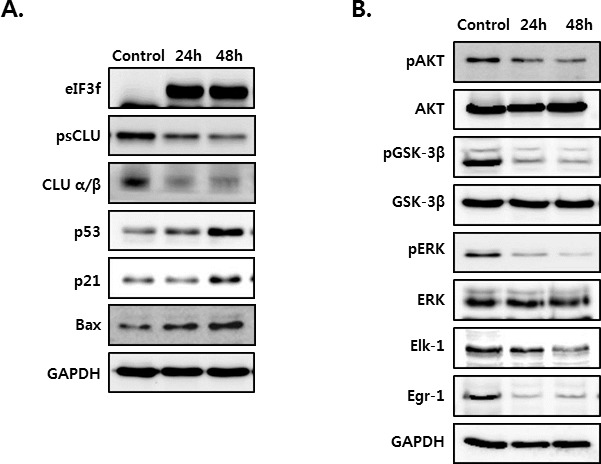
eIF3f overexpression activated p53 and inhibited Akt and ERK signaling HeLa cells were transiently transfected with pcDNA4-eIF3f, and harvested at 24-h intervals. The expression of eIF3f, psCLU, α/β-CLU, p53, p21, and Bax **A.** and phosphorylated and total Akt, GSK-3β, ERK, Elk-1, and Egr-1 **B.** were detected using Western blotting. GAPDH was used as the reference for equal protein loading.

To assess whether the overexpression of eIF3f inhibits the function of CLU, we next measured the expression of several proteins that induce apoptosis. HeLa cells were cultured in the absence or presence of eIF3f for 24 and 48 h. As shown in Figure 6, eIF3f transfection in HeLa cells upregulated the expression of the apoptotic proteins p53, p21, and Bax (Figure 6A). Conversely, depletion of sCLU caused by eIF3f overexpression reduced the phosphorylation of both Akt and ERK compared with control HeLa cells. In addition, eIF3f decreased the phosphorylation of GSK-3β and expression of Elk-1 and Egr-1, downstream factors of Akt and ERK, respectively (Figure 6B). This suggests that eIF3f overexpression suppressed the signaling pathways activated by sCLU. Therefore, eIF3f inhibits cancer cell growth and induces apoptosis by inhibiting sCLU function.

### eIF3f increases the stability of p53

**Figure 7:**
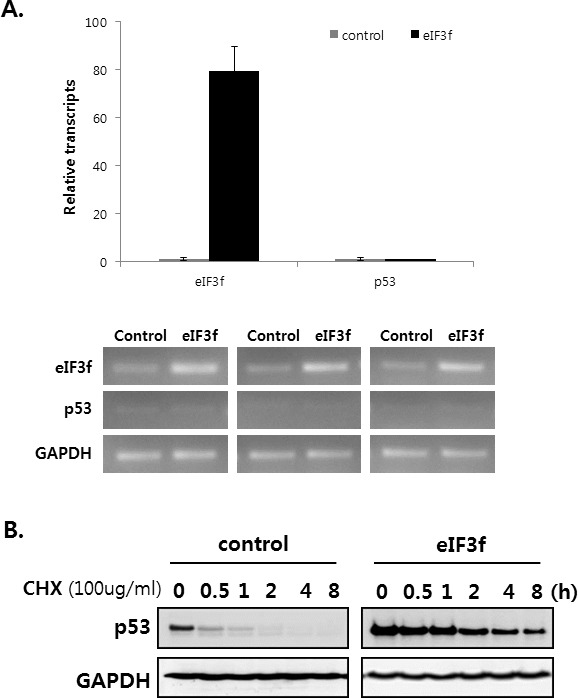

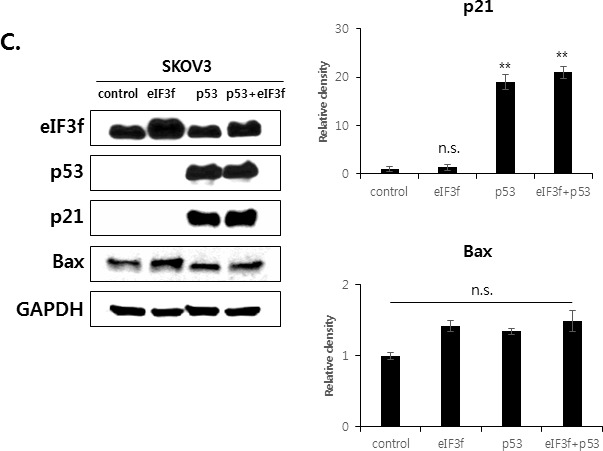
eIF3f stabilizes p53 protein HeLa cells were transfected with pcDNA4-eIF3f. **A.** The levels of *p53* and *eIF3f* were then analyzed using qRT-PCR 48 h after transfection. Levels were normalized against those of *GAPDH* mRNA and are reported as means ± SD. **B.** Twenty-four hours after transfection, cells were incubated with CHX (100 μg/ml) for the indicated time points, and p53 stability was analyzed by Western blotting with p53 and GAPDH antibodies. **C.** p21 and Bax are regulated by p53-dependent or -independent pathways. SKOV3 p53^−/−^ cells were transfected transiently with pcDNA3-p53 and pcDNA4-eIF3f. Total cell lysates were then analyzed by Western blotting 48 h after transfection. The graphs on the right show the intensity of the bands, as quantified using Image J software.

A previous result revealed that eIF3f increases p53 protein levels (Figure 6A). Therefore, we assessed how eIF3f inhibits p53 expression using qRT-PCR and cycloheximide (CHX) assays. We first measured whether eIF3f affects p53 levels. qRT-PCR revealed that eIF3f did not stimulate the upregulation of p53 mRNA (Figure 7A), suggesting that eIF3f does not inhibit p53 levels. Therefore, we hypothesized that eIF3f might stabilizes p53, because sCLU inhibits the p53 stability [31]. Therefore, HeLa cells were treated with cycloheximide to inhibit protein translation, and then the p53 degradation rate was compared between eIF3f- and control-transfected cells. As shown in Figure 7B, eIF3f increased and stabilized the levels of endogenous p53. These results suggest that eIF3f promotes the tumor suppressor function of p53 by increasing the stability of p53 protein.

We next defined the relationship among p53, p21, and Bax during eIF3f-induced apoptosis. SKOV3 p53^−/−^ cells were transfected with eIF3f or p53. Transfection with eIF3f resulted in the accumulation of p21 in p53-transfected cells, whereas Bax was activated in both p53-transfected and un-transfected cells. In addition, Bax expression was increased to a greater extent in cells transfected with both p53 and eIF3f compared with p53 alone. Western blotting then revealed that the upregulation of p21 after eIF3f overexpression was p53-dependent, whereas Bax activation was both p53-dependent and -independent (Figure 7C).

### eIF3f-induced apoptotic signal is related to inhibition of CLU

**Figure 8:**
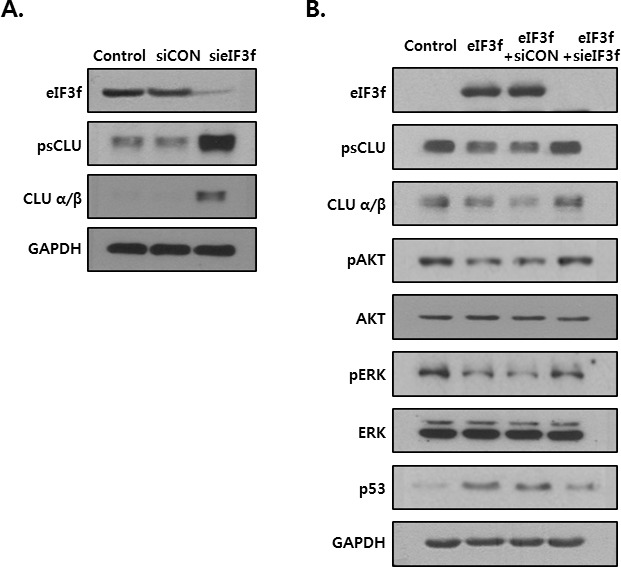
eIF3f-induced apoptotic signal is related to inhibition of CLU **A.** HEK293a cells were transfected with scramble siRNA (siCON) or eIF3f siRNA (sieIF3f) for 72h. Whole cell extracts were analyzed by Western blotting. **B.** HeLa cells were transiently transfected with pcDNA4, pcDNA4-eIF3f, siCON, and sieIF3f, respectively. Total cell lysates were then detected by Western blotting. GAPDH was used as the reference protein for equal loading.

The previous figures show that eIF3f inhibited CLU expression and induced apoptotic signal. So, we examined whether this effect is especially mediated by inhibition of CLU. Knockdown of eIF3f in the HEK293a cells increased the CLU expression at 72h post transfection (Figure 8A). As shown in Figure 8B, CLU expression was diminished in eIF3f overexpressed HeLa cells, wheras it was restored to its original expression by transfection of eIF3f siRNA. Furthermore, expression of downstream factors of CLU was also returned to their intrinsic expression, together. These results suggest that anti-proliferative effect of eIF3f is caused by inhibition of CLU.

### eIF3f inhibits tumor growth *in vivo*

**Figure 9:**
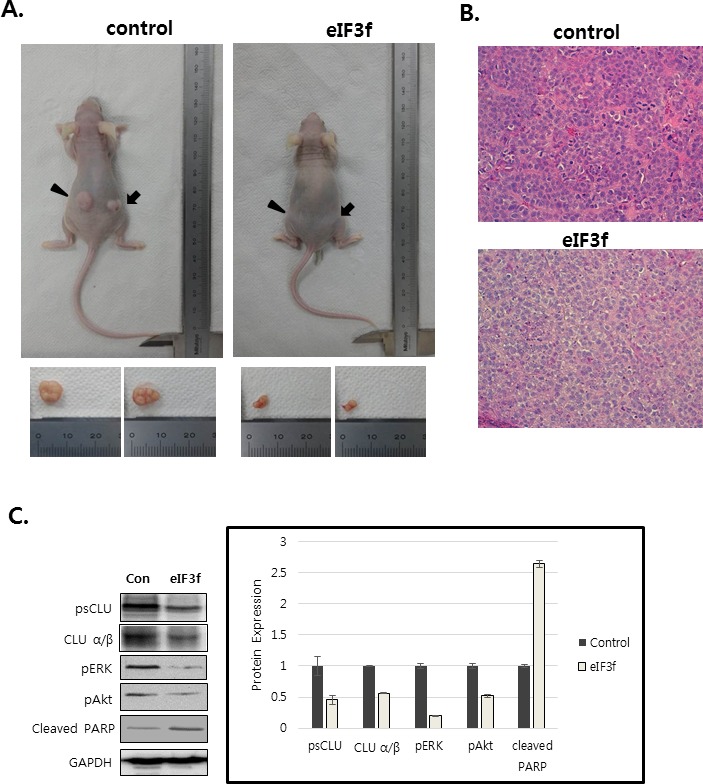

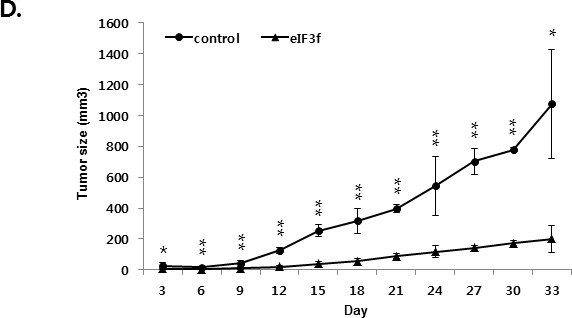
eIF3f inhibits tumor growth *in vivo* Control or eIF3f-transfected HeLa cells (2.5 × 10^6^ per mouse) were implanted into nude mice. **A.** The images show representative samples on day 33. The arrow indicates the injection sites. **B.** Paraffin sections of eIF3f treated and control tumors were stained with hematoxylin-eosin(H&E) **C.** Soluble protein extracts from xenograft mice were subjected to immunoblotting for the indicated proteins (CLU, pAkt, pERK, cleaved PARP). **D.** The growth curves show the calculated tumor sizes in mice of each group (*n* = 5 to 7 per group), and data are presented as means ± SD at each time point.

We next explored whether eIF3f inhibits tumor growth *in vivo*. Exponentially growing *HeLa* cervical cancer cells were transfected transiently with eIF3f expression or control vector, and the cells were injected subcutaneously into immune-deficient BALB/c nude mice. Animals (5-7 per group) were then monitored for tumor growth and morphology for 33 days. Figure 9A shows that the tumor mass in mice transfected with pCDNA3-eIF3f was diminished significantly compared with tumors in mice transfected with control vector. Collectively, these results suggest that eIF3f had obvious antitumor effects. Paraffin sections of tumor tissue were stained by H&E for histological analysis. The control showed high-grade carcinoma with an irregular cell distribution. In contrast, eIF3f treated tumor exhibited large areas of apoptotic cells (Figure 9B). We next investigated the inhibitory effect of eIF3f on CLU expression and Akt and ERK signaling pathway in tumor tissues harvested from control and eIF3f treated mice. eIF3f transfection was observed to significantly decrease the psCLU and α/β CLU expression and inhibit Akt and ERK phosphorylation. And the cleaved PARP, a apoptosis marker level was increased (Figure 9C). Collectively, these results clearly demonstrate that eIF3f can be a potent tumor suppressor in this animal model.

## DISCUSSION

Changes in CLU expression were related to tumor stage and grade [17]. The upregulated expression of CLU was associated with highly aggressive breast carcinoma [9, 32] and with tumor progression and recurrence in bladder cancer [33]. The overexpression of CLU was detected in metastatic ovarian and cervical cancer compared with matched normal tissues [15, 34]. In addition, the transfection of CLU into human renal carcinoma cells enhanced cancer cell survival and metastatic potential [35, 36]. Moreover, *CLU* gene silencing in human bladder and prostate cancers inhibited growth and increased their chemosensitivity [33, 37]. Although a number of studies analyzed CLU expression in relation to tumorigenesis, the details of its biological roles and regulatory mechanisms in human cancer cells remain unknown. Therefore, understanding the mechanism of action of CLU in cancer cells is essential for the development of novel effective cancer treatments.

This study shows the crucial function of eIF3f as a novel potent antitumor agent that inhibits the function of anti-apoptotic factor CLU. Herein, we suggest a possible mechanism behind the eIF3f-induced inhibition of tumor growth that involves the direct binding of eIF3f to CLU, which inhibits sCLU-induced activation of Akt and ERK signaling.

eIF3f is a subunit of the mammalian eIF3 complex, which binds to the 40S ribosome and promotes the binding of methionyl-tRNA and mRNA [21]. However, loss of the *eIF3f* gene has been observed in melanoma and pancreatic cancer, which results in the downregulation of eIF3f [26, 27]. Similarly, several previous studies reported that eIF3f expression was reduced in cervical and ovarian cancer by ~60-70%. Moreover, increased eIF3f inhibits translation and cell growth and induces apoptosis in melanoma and pancreatic cancer cells [25]. These reports suggest that the downregulation of eIF3f might be the major cause of cancer pathogenesis. However, its function in cancer remains unclear. The current results revealed that eIF3f expression was decreased dramatically in human pancreatic, cervical, and ovarian cancer cells (Figure 2), consistent with the above mentioned studies. In addition, eIF3f reduces cell growth and increases apoptosis significantly in HeLa and BxPc-3 cells (Figure 3). However, interestingly, these effects were minimal in cell lines that express low levels of endogenous CLU (data not shown). Therefore, because eIF3f interacts with CLU, the effect of eIF3f on cell proliferation requires the expression of CLU.

To assess how eIF3f inhibits CLU activity, we generated several deletion constructs and analyzed the specific sites responsible for the binding between eIF3f and CLU. Data revealed that eIF3f binds with the α-chain of sCLU encoded by amino acids 1-227 (Figure 4). Generally, sCLU is expressed highly in cancer, in which it plays roles as a pro-survival factor in tumor progression and metastasis [38, 39, 40]. sCLU is generated as an unglycosylated precursor protein (psCLU) that is cleaved proteolytically into α- and β-subunits, which are then held together by disulfide bonds, and the protein is secreted [1, 3]. Therefore, we hypothesized that eIF3f disrupts the cleavage of sCLU or the interaction between the α- and β-subunits by interacting directly with the α-subunit. Indeed, increasing the expression of eIF3f reduces the amount of sCLU precursor, α/β-CLU expression, and sCLU secretion from the cell (Figures 5, 6A). These results suggest that eIF3f activates the structural modification of sCLU into its mature form, which results in decreased sCLU expression and secretion. In addition, the *CLU* promoter is activated by IGF-1R/Src/MAPK signaling [41]. Decreased sCLU levels in cells overexpressing eIF3f inhibit ERK activation and Egr-1 expression (Figure 6B). These data suggest that depleted Egr-1 expression reduces psCLU expression by inhibiting *CLU* promoter transcriptional activity.

In osteosarcoma U2-OS cells, sCLU depletion induced Bax activation by suppressing Bcl-2 protein and stabilizing p53, which leads to the activation of *p21* and *Bax* genes [31]. Moreover, sCLU regulates mitochondrial apoptosis by stabilizing the cytosolic Ku70-Bax protein complex. Bax is maintained in an inactive form in the cytosol by binding to Ku70. sCLU stabilizes the Ku70-Bax complex and inhibits the conformational change in Bax and its subsequent translocation to the mitochondria [31, 42]. In addition, α-chain of sCLU binds directly with activated Bax and prevents the oligomerization of Bax, thereby suppressing the intrinsic apoptotic pathway [43]. Consistent with previous reports, the current study demonstrated that the overexpression of eIF3f enhanced p53, p21, and activated Bax levels in a time-dependent manner in HeLa cervical cancer cells (Figure 6A). In addition, eIF3f elevated p21 in only a p53-dependent manner in SKOV3 p53^−/−^ cells but elevated Bax in both p53-dependent and -independent manners (Figure 7C). Therefore, we hypothesized that eIF3f disrupts the stability of the Ku70-Bax complex because eIF3f binds to sCLU to deplete the expression of sCLU. However, it remains unclear whether eIF3f disrupts this complex. Furthermore, the present study identified a previously unrecognized pathway that links sCLU depletion to p53 activation. Interestingly, eIF3f did not alter the levels of p53 mRNA but stabilized p53 protein (Figure 7A, 7B). However, understanding the mechanism behind these effects requires further investigation.

The current data in HeLa cells revealed that transfection with eIF3f reduced the phosphorylation of Akt and ERK and inhibited the downstream pathways pGSK-3β, Elk-1, and Egr-1. However, there are several pathways downstream of Akt and ERK. Therefore, the identification of other effects requires further study. Recently we reported PACAP as a CLU binding protein [44]. Interestingly, eif3f interacts with α-chain but PACAP binds with β-chain of CLU. And PACAP showed weaker but similar CLU suppression function with eif3f. So the further study of the regulatory mechanism of eif3f and PACAP on CLU may valuable.

In conclusion, the current study revealed a novel function of eIF3f as a regulator of the survival factor sCLU in human tumors. The data also suggest that eIF3f inhibits cancer cell growth and induces apoptosis by blocking the function of sCLU, thereby suppressing the inactivation of p53 and Bax as well as the Akt and ERK pathways. This newly discovered mechanism might lead to the future development of effective CLU-targeting therapies for cancer patients

## MATERIALS AND METHODS

### Cell culture and transfection

HEK293a, HeLa, BxPc-3 and SKOV3 cell lines were obtained from the American Type Culture Collection (ATCC). All cell lines were incubated at 37°C in a humidity 5% CO2 incubator, and cultured in each recommended medium supplemented with 10% fetal bovine serum (FBS, WelGENE) and 1% Amphotericin B/Streptomycin/Penicillin (Gibco). Cell transfection was performed using the X-tremeGENE HP reagent (Roche Applied Science) according to the manufacturer's instructions.

### Construction of plasmids

pOTB7-eIF3f plasmid was provided from Korea Human Gene Bank, Medical Genomics Research center, KRIBB, Korea. eIF3f expression vectors were constructed by cloning full-length eIF3f into pcDNA4 and pcDNA3-flag vector using *EcoR I* and *Xho I* restriction sites. pIRES-CLU plasmid was provided by Dr.Saverio Bettuzzi, university of Parma, Italy and inserted in pcDNA3.1 vector using *BamH I* and *Xho I* restriction sites.

### Yeast two-hybrid assay

The EGY48 yeast strain was used in present study and Matchmaker LexA Two-Hybrid system (Clontech, Palo Alto, CA) was used to perform the yeast two-hybrid assay according to the manufacture's instructions. The wild type and deleted CLU were amplified by PCR and cloned into pGilda vector using *BamH I* and *Xho I* restriction sites and used as baits. Human cDNA library was inserted in pB42AD prey vector for the yeast-two hybrid screening. Also, the wild type and deleted eIF3f were amplified by PCR and cloned into pB42AD prey vector between *EcoR I* and *Xho I* restriction sites for the growth and Δ-galactosidase assay. The primers used for amplication are shown in the Table 1. Bait and prey vectors were co-transformed in EGY48 yeast strain and transformants were grown for 3 days at 30°C on plates in dropout media lacking uracil, histidine and tryptophan. Positive colonies were confirmed by growth and β-galactosidase assay on plates lacking uracil, histidine, tryptophan and leucine or containing X-gal, respectively.

**Table 1:** Primers used in cloning of yeast two-hybrid assay

Gene	Location	Primer sequences	Restriction site
CLU	Full (1-449)	F 5′-ATGCGGATCCATGATGAAGACTCTGCTG-3′	*BamHI*
		R 5′-ATGCCTCGAGTCACTCCTCCCGGTGCTT-3′	*XhoI*
	34-449	F 5′-ATGCGGATCCCGATGTCCAATCAGGGAAGT-3′	*BamHI*
		R 5′-ATGCCTCGAGTCACTCCTCCCGGTGCTT-3′	*XhoI*
	1-227	F 5′-ATGCGGATCCGCATGATGAAGACTCTGCTG-3′	*BamHI*
		R 5′-ATGCCTCGAGTAGCGGACGATGCGGGA-3′	*XhoI*
	228-449	F 5′-ATGCGGATCCGCATGAGCTTGATGCCCTTC-3′	*BamHI*
		R 5′-ATGCCTCGAGTCACTCCTCCCGGTGCTT-3′	*XhoI*
eIF3f	Full (1-358)	F 5′-ATGCGAATTCATGGCCACACCGGCG-3′	*EcoRI*
		R 5′-ATGCCTCGAGTCACAGGTTTACAAG-3′	*XhoI*
	1-170	F 5′-ATGCGAATTCATGGCCACACCGGCG-3′	*EcoRI*
		F 5′-ATGCCTCGAGTCAGATGAGCTCATT-3′	*XhoI*
	170-358	F 5′-ATGCGAATTCATGCTGGGCTGGTAC-3′	*EcoRI*
		R 5′-ATGCCTCGAGTCACAGGTTTACAAG-3′	*XhoI*
	248-358	F 5′-ATGCGAATTCATGGGAGTTGACCTG-3′	*EcoRI*
		R 5′-ATGCCTCGAGTCACAGGTTTACAAG-3′	*XhoI*

### Co-immunoprecipitation (Co-IP)

Cells were lysed in NP-40 lysis buffer (20mM Tris-HCl pH8.0, 150mM NaCl, 1% Nonident P-40, 1mM PMSF) for 30min on ice. Extracts were centrifuged at 13,000rpm for 10min at 4°C, and the protein concentration was measured using the Bradford assay. Each cell lysate (1.5mg) was incubated with CLU polyclonal antibody (Santa cruz) or flag monoclonal antibody (Sigma) for overnight at 4°C. Following incubation, protein was immunoprecipitated using protein A/G agarose beads (Santa cruz) for 3hr at 4°C with gently rotation. The immunoprecipitates was washed three times with lysis buffer and boiled in 40°C of 1X SDS sample buffer for 5min at 95°C. After centrifugation, the supernatant was analyzed by Western blot.

### Immunocytochemistry (ICC)

Cells were grown on 18mm diameter cover glass (Marienfeld). After 48hr incubation, the cells were rinsed twice with 1X PBS and fixed and permeabilized in methanol-acetone mixture (1:1) for 7min at −20°C. Fixed cells were blocked with 5% BSA/PBS-T (PBS, 0.2% Tween-20) for 1hr at room temperature, and then incubated with CLU polyclonal antibody (1:300 dilution) and eIF3f polyclonal antibody (1:300 dilution) for overnight at 4°C. The cells were washed three times with 1X PBS for 5min each and incubated with alexa fluor 488 goat anti-rabbit IgG (Green) and alexa fluor 568 donkey anti-goat IgG (Red) antibody (Invitrogen, 1000:1 dilution) in darkness for 1hr at room temperature. Finally, the cells were counterstained with 1μg/ml DAPI for 1min at room temperature and mounted on slides. The signals and co-localization were detected using the confocal fluorescene microscopy.

### Quantitative real-time PCR (QRT-PCR)

All RNA from cells was extracted by using TRIZOL reagent (Invitrogen), and the cDNA was synthesized with 1 μg of total RNA using Iscript^TM^ select cDNA synthesis kit (Bio-Rad). Real-time PCR was performed using 2X SYBR Green Supermix (Bio-Rad) in MiniOpticon^TM^ system. All experimental procedures were carried out according to the manufacturer's standard protocols. The PCR reaction mixture (total volume of 20 μl) was consisted of the following: 5ng of cDNA, 10 μl of 2X SYBR Green Supermix and 5pM of both forward and reverse primers. The PCR conditions were: an initial denaturation step of 95°C for 5min, followed by 40 cycles of 95°C for 10sec, 55~60°C for 15sec and 72°C for 20sec. The target and control genes amplification was conducted in seperated tubes. The reactions were repeated in triplicate. Relative quantification was calculated as the target gene/GAPDH ratio, and gene expression was analyzed with MJ Opticon Monitor analysis software (Bio-Rad). PCR products were electrophoresed by 2% agarose gel with ethidium bromide. Primers for eIF3f were forward 5′-TGACAGTGAAATACGCGTAC-3′ and reverse 5′-GTCACTTGAGAGTCCAATCAC-3′. Primers for p53 were forward 5′-GTTCCGAGACGTGAATGAGG-3′ and reverse 5′-TTTTATGGCGGGACGTAGAC-3′. Primers for GADPH were forward 5′-GACTCCACTGGCGTCTTCAC-3′ and reverse 5′-GTTCACACCCATGACTAACA-3′.

### Cell proliferation assay

The cell viability was examined by the Cell Counting Kit-8 (CCK-8) assay. HeLa and Bxpc-3 cells were seeded in 96-well plates and transiently transfected. CCK-8 reagent was treated to each well and incubated for 1hr at 24hr intervals. The O.D value was measured at 450nm in triplicate.

For colony formation assay, transfected cells were grown at 1,000 cells per 100mm cell culture dish in triplicate. Then, the cells were incubated to allow colonies to form for 2weeks. After the incubation, cells were washed twice with 1X PBS, fixed in 4% paraformaldehyde (Sigma) for 15min, and stained with 0.5% crystal violet (in D.W, Sigma) at room temperature for 15min. The plates were rinsed with water and total colony number was counted.

### Annexin V/PI staining

The cells were plated into 6-well plates and transfected with expression vectors. Transfected cells were havested with Detachin solution at different time point and pelleted by centrifugation. Each of the cells was washed once with 1X PBS and resuspended in 100 μl of Annexin V binding buffer. Annexin V-FITC and Hoechst 33342 were then added to cell suspension. After the incubation at 37°C for 15min, cells were washed with Annexin V binding buffer, stained with 2 μl of PI (propodium iodide, 500 μg/ml) and immediately analyzed with NucleoCounter^®^ NC-3000^TM^.

### Preparation of protein extracts

Cell pellets were resuspended in RIPA buffer (50mM Tris-Cl pH 7.5, 150mM NaCl, 1% NP-40, 0.5% sodium deoxycholate, 0.1% SDS, 5mM PMSF) for 30min on ice. The extracts were centrifuged at 13,000rpm for 10min at 4°C, and the protein concentration was determined using the Bradford assay.

Secreted protein in medium was prepared using the TCA (trichloroacetic acid) precipitation. 1ml of culture medium was precipitated with 250 μl of 100% TCA for 30min on ice. Pellet was washed twice with pre-cooled acetone, dried at 95°C for 5min, and resuspended in 100 μl of 1X SDS sample buffer by neutralizing with 1M Tris-HCl (pH 8.0). After centrifugation, the supernatant was used for Western blot.

### Western blot

Protein extracts were prepared as described above, then separated by SDS-PAGE using 8 to 15% polyacrylamide gel and transferred onto a nitrocellulose membrane (Bio-Rad, 0.45μM). The membranes were blocked for 1hr in 5% skim milk and incubated at 4°C for overnight with specific primary antibody. Antibodies against CLU (sc-6419), p53(sc-6243), p21 (sc-397), Bax (sc-493), pERK (sc-7383), Elk-1 (sc-355), Egr-1 (sc-189), GSK-3β (sc-8257) and GAPDH (sc-25778) were purchased from Santa Cruz Biotechnology. Antibodies against p-Akt (#4060), pGSK-3β (#9331) and cleaved PARP (#9541) were purchased from Cell signaling. Antibodies against eIF3f (638202), Akt (1085-1) and flag (F1804) were purchased from Biolegend, EPIT-MICS and Sigma aldrich, respectively. Following primary antibody incubation, the membranes were washed three times for 5min in 1X TBS-T, incubated for 1hr with horseradish peroxidase-conjugated secondary antibody. Immunoreactivity was detected using ECL chemiluminescent solution (advansta) and exposed by Chemidoc (Bio-Rad).

### Animal experiments

All surgical procedures and care administered to the mice were in accordance with institutional guidlines. Six-week-old male immune-deficient BALB/c nu/nu mice were purchased from Daehan Biolink Co. (Chungbuk, Korea). HeLa cells were seeded into 150mm cell culture dish and transfected with expression vector. After 24hr incubation, 2.5×10^6^ of cells were subcutaneously injected into the back of mice. Tumor sizes were calculated every three days: ab^2^/2 (a is the short axis and b is the long axis). Mice were killed on day 33 and tumors were excised.

### Knockdown of eIF3f expression by siRNA

eIF3f siRNA and scramble siRNA were synthesized by siRNA kit (Ambion) according to the manufacturer's protocols. The target sequence of the eIF3f is: 5′-AAAGGTGTCAGCTGACAATAC-3′. Scramble siRNA oligonucleotide was included in the kit. eIF3f siRNA or scramble siRNA was transfected in to the cells using the Lipofectamine RNAi/Max reagent (Invitrogen) according to the manufacturer's instructions. Cells were harvested and cell extracts were analyzed by Western blot.

### Statistical analysis

All statistical analysis was performed using the Excel software (Microsoft Excel, 2010). Differences between experimental groups were compared by ANOVA and *t*-test. *P* value < 0.05 was considered statistically significant. Each experiment was repeated more than three times and the data were presented as mean±SD.
