# Correction: eIF3f reduces tumor growth by directly interrupting clusterin with anti-apoptotic property in cancer cells

**DOI:** 10.18632/oncotarget.18267

**Published:** 2017-05-29

**Authors:** Ji-Yeon Lee, Hyun-Ji Kim, Seung Bae Rho, Seung-Hoon Lee

**Present:** The grant number information is incorrect in the Acknowledgments.

**Correct:** The proper grant information appears below:

**ACKNOWLEDGMENTS**

This research was supported by Basic Science Research Program through the National Research Foundation of Korea (NRF) funded by the Ministry of Education, Science and Technology (2014R1A1A2055411).

Original article: Oncotarget. 2016; 7:18541-18557. doi: 10.18632/oncotarget.8105

